# The evolving treatment landscape for hereditary angioedema in Sweden

**DOI:** 10.3389/fimmu.2026.1828443

**Published:** 2026-07-29

**Authors:** Sabrina Meisgen, Franziska Fischer, Yinglei Li

**Affiliations:** 1 CSL Behring AB, Stockholm, Sweden; 2 Quantify Research AB, Stockholm, Sweden; 3 CSL Behring, King of Prussia, PA, United States

**Keywords:** dispensation patterns, hereditary angioedema (HAE), long-term prophylaxis (LTP), on-demand treatment (ODT), prescribed drug register

## Abstract

**Introduction:**

Hereditary angioedema (HAE) is a rare genetic disorder, characterized by unpredictable, potentially life-threatening edema attacks. Treatment options include on-demand treatment (ODT) and long-term prophylactic (LTP) therapy. This study describes the HAE treatment landscape in Sweden, focusing on the uptake of recently reimbursed modern LTP therapies and patterns of ODT dispensation as an indirect proxy for breakthrough attacks.

**Methods:**

This nationwide, longitudinal, registry-based study utilized data from the National Prescribed Drug Register in Sweden describing all dispensed drugs with approved indications for HAE between January 2020–May 2025.

**Results:**

During 2024, 245 patients received HAE-specific drugs, with one-third dispensing LTP. Among patients with ≥12 months on modern LTP (n=69) who had at least one ODT dispensation event during their most recent 12 months of treatment (n=46/59 for lanadelumab; n=8/9 for berotralstat), the mean number of ODT packages dispensed—in addition to emergency stock ODT—was 4.3 per patient on lanadelumab and 6.5 per patient on berotralstat. Overall, 22% (n=15/69) of patients receiving modern LTP did not dispense ODT medication during their most recent 12-months of treatment. In total, 22% of patients (n=24/108) with at least one lanadelumab or berotralstat dispensation stopped dispensing modern LTP and switched to alternative LTP or ODT-only treatment options.

**Discussion:**

This study provides valuable insights into real-world dispensation patterns of HAE drugs in Sweden. Despite the availability of modern LTP therapies, there remains relatively high ODT dispensation events, suggesting ongoing unmet need and underscoring the importance of optimizing prophylactic strategies to achieve total disease control.

## 1 Introduction

Hereditary angioedema (HAE) is a rare genetic disorder that arises from low plasma levels of functional C1-esterase inhibitor (C1INH) (HAE-C1INH Type 1) or normal/elevated plasma levels of dysfunctional C1INH (HAE-C1INH Type 2), resulting in uncontrolled activation of the kallikrein-kinin system leading to the excess production of bradykinin, increased vascular permeability and subsequent angioedema attacks (1, 2). Additionally, patients with normal C1 inhibitor levels and activity (HAE-nC1INH) may also experience HAE attacks, although its prevalence is less common (3). Recent clinician-reported survey data from the United States found that around 77–81% of patients with HAE are diagnosed with HAE type 1 or type 2, and 19–23% are diagnosed with HAE-nlC1INH (4). Angioedema attacks are unpredictable, recurrent, and can be potentially life-threatening (1).

The international World Allergy Organization (WAO)/European Academy of Allergy and Clinical Immunology (EAACI) guideline for the management of HAE, revised in 2021, defines the ultimate treatment goals as achieving complete disease control—meaning the absence of angioedema attacks—and normalization of patients’ lives (1). At present, these goals can only be met through long-term prophylaxis (LTP) treatment, which aims to prevent attack occurrence. In contrast, on-demand treatments (ODTs) are recommended for managing acute attacks and should be administered as early as possible to minimize their impact (1). Accordingly, the guideline recommends that all patients carry sufficient ODT to treat at least two attacks (1).

The WAO/EAACI guideline recommends that all patients should be evaluated for LTP at every clinical visit, with visits occurring at least once per year (1). These evaluations should consider disease activity, overall disease burden, level of disease control, and patient preference; however, not all patients ultimately receive LTP (1). Recent multinational surveys indicate that only 50–64% of patients receive LTP, with approximately half continuing to rely solely on ODT (5, 6). The selective use of LTP could reflect a balance between clinical benefit, patient preference, economic feasibility, and practical considerations (7, 8). Moreover, breakthrough attacks remain common even among patients on LTP, contributing to persistent disease burden, impaired quality of life, mental health challenges, and reduced productivity (5, 6).

In Sweden, the HAE treatment landscape has evolved over the years, providing patients access to a broad range of ODT and LTP treatment options (9). A previous report investigating trends in treatments with disease-specific and interfering drugs in patients with HAE in Sweden from 2005–2019—before modern LTP therapies became reimbursed—revealed high utilization of attenuated androgens and increasing use of C1 inhibitor for prophylaxis (9). The common use of ODT suggested suboptimal disease control in many patients, highlighting the need for increased focus on prophylactic strategies (9).

At the time the previous report was published (9), LTP treatment in Sweden was entering a paradigm shift, marked by the emergence of orally administered small-molecule inhibitors and subcutaneously administered monoclonal antibodies targeting plasma kallikrein.

This study aims to evaluate how the availability of these modern LTP treatment options has reshaped the national treatment landscape, with particular emphasis on whether their adoption has influenced reliance on ODT medication in the treatment of breakthrough attacks.

## 2 Methods

### 2.1 Data source

This nationwide, registry-based study utilized data from the Swedish National Prescribed Drug Register (Läkemedelsregistret) pertaining to all dispensed drugs with approved indications for HAE treatment (Anatomical Therapeutic Chemical [ATC] code B06AC and subcodes B06AC01–B06AC06) in Sweden and dispensed at pharmacies between January 2020–May 2025. The registry data included product name and strength, ATC codes, date of dispensation, and number of packages per dispensing event for products under ATC code B06AC01 (plasma-derived C1 inhibitor).

### 2.2 Study population

Patients with at least one dispensation event of any HAE-specific medication during the study period (January 2020–May 2025) were included in the study population.

### 2.3 Outcomes measures

#### 2.3.1 Overall HAE drug dispensation analysis

The number of unique patients prescribed any HAE-specific medication, and the number of new patients prescribed modern LTP treatments lanadelumab or berotralstat were evaluated from January 2020 to May 2025. Subcutaneous plasma-derived C1INH replacement is not reimbursed for prophylactic use in Sweden and thus was not included in this analysis.

#### 2.3.2 Modern LTP treatment and ODT dispensations

**Figure 1:**
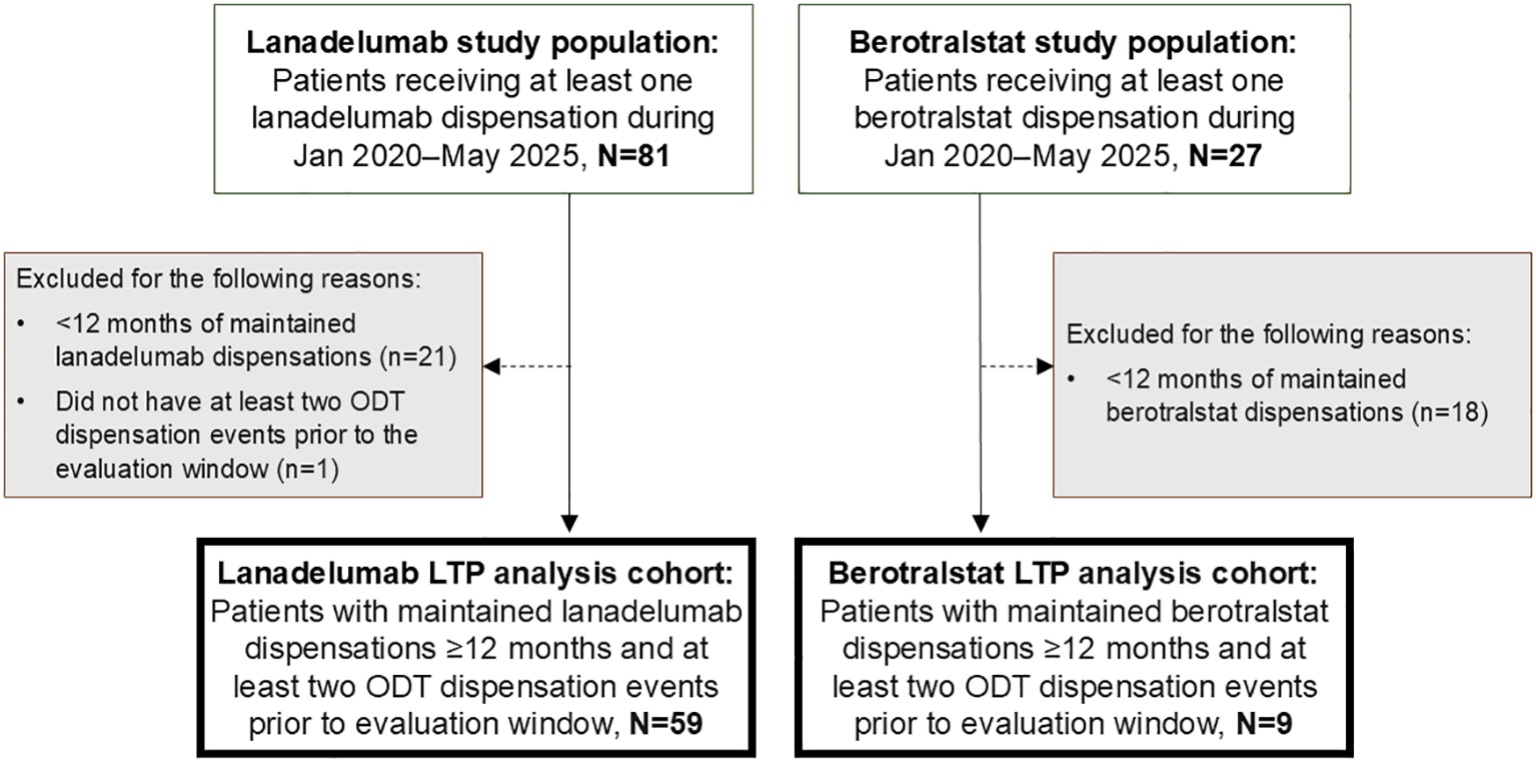
Population for analysis: ODT dispensations among patients receiving lanadelumab or berotralstat LTP. Patients with at least one lanadelumab or berotralstat dispensation event between January 2020 and May 2025 were first identified, and those with dispensations for at least 12 months were included in the analysis. Patients with <12 months lanadelumab or berotralstat dispensations and who had less than two ODT dispensation events prior to the evaluation window were excluded. All patients included in the analysis dispensed ODT medication prior to entering their 12-month observation window, equivalent to the recommended stock medication (1). LTP, long-term prophylaxis; N, number; ODT, on-demand treatment.

Patients who had lanadelumab or berotralstat prophylaxis therapy for at least 12 months were included in the analysis to ensure sufficient follow-up for meaningful analyses (**Figure 1**). In absence of information on the number of dispensed packages for ATC code B06AC05 (lanadelumab) and B06AC06 (berotralstat), maintained lanadelumab or berotralstat prophylaxis therapy was defined based on dispensation patterns in accordance with Swedish pharmacy regulations i.e., maximum of three-month medication supply per dispensation event or several dispensation events adding up to the medication supply for a three-month period, in combination with product-specific package size available in Sweden. For lanadelumab therapy, this corresponds to patients having no more than 10 months between two dispensation events and/or at least one dispensation event within the 10 months prior to the May 2025 data cut. For berotralstat therapy, this corresponds to patients having no more than four months between two dispensation events and/or at least one dispensation event within the four months prior to the May 2025 data cut.

For each patient on prophylactic therapy, ODT dispensation events were evaluated during the patient’s individual most recent 12 months of LTP treatment corresponding to the recommended annual HAE treatment evaluation timeframe. All patients included in this analysis had at least two ODT dispensation events prior to entering the 12-month observation window, equivalent to the emergency stock medication patients should have at home in accordance with current treatment guidelines (1).

### 2.4 Imputation for missing package information

As number of ODT packages was the outcome to analyze, an imputation procedure was applied when information on the number of ODT packages were unavailable. ODT package information was available for all dispensations made under the ATC-code B06AC01 revealing that one package was dispensed per every dispensation event in the dataset. For dispensation events under the ATC-code B06AC02, package data were missing. For these records, the number of packages was imputed using a scenario-based approach. The conservative scenario assumed one package per dispensation event, whereas the moderate scenario assumed two packages per event. Dispensation of more than two packages per event was not considered plausible based on clinical practice in Sweden (10).

### 2.5 Statistical analysis

Descriptive statistics were used to summarize dispensation patterns. No inferential hypothesis testing was performed, in accordance with the descriptive nature of this registry-based utilization study. All statistical analyses were performed using R software (version 4.2.3).

## 3 Results

### 3.1 HAE drug dispensation patterns in Sweden

The annual number of unique patients dispensing HAE-specific medications increased steadily over the study period, rising from 170 patients in 2020 to 188 in 2021, 205 in 2022, 233 in 2023, and 245 in 2024. Between January and May 2025 (data cut), a total of 195 patients made at least one HAE-specific drug dispensation. The Swedish National Prescribed Drug Register does not detail disease-specific information. As such, HAE subtypes could not be determined within the study population.

Reimbursed ODT medications in Sweden (January 2020–May 2025) included plasma-derived C1INH (pdC1INH intravenous (IV); Berinert and Cinryze), icatibant, and conestat alpha, with ceased dispensations after 2020 (**Table 1**). The number of patients who dispensed IV pdC1INH remained stable during the study period, whereas the number of patients dispensing icatibant increased (**Table 1**). Among the 245 patients with HAE-specific drug dispensations during calendar year 2024, 110 dispensed IV pdC1INH and 189 dispensed icatibant; referring to patients on ODT regimen and those with ODT use while on LTP treatment regimen (**Table 1**).

**Table 1:** HAE drug prescription patterns in Sweden: number of unique patients with HAE-specific drug dispensations per time period.

HAE drug	2020	2021	2022	2023	2024	January–May 2025
	Patients (n, %)	Patients (n, %)	Patients (n, %)	Patients (n, %)	Patients (n, %)	Patients (n, %)
IV pdC1INH^*†^	117 (69%)	114 (61%)	120 (59%)	112 (48%)	110 (45%)	73 (37%)
Icatibant	121 (71%)	138 (73%)	152 (74%)	174 (75%)	189 (77%)	124 (64%)
Conestat alpha	1 (0.6%)	0 (0%)	0 (0%)	0 (0%)	0 (0%)	0 (0%)
Lanadelumab	2 (1%)	5 (3%)	36 (18%)	54 (23%)	68 (28%)	71 (36%)
Berotralstat	0 (0%)	1 (0.5%)	16 (8%)	13 (6%)	14 (6%)	9 (5%)

Number of unique patients with HAE-specific drug dispensations per time period (2020 N = 170; 2021 N = 188; 2022 N = 205; 2023 N = 233; 2024 N = 245; and until May 2025 N = 195) Patients can dispense more than one product per period. *Two patients may have dispensed IV pdC1INH (specifically Cinryze) for prophylactic use to prevent HAE attacks. ^†^All IV pdC1INH (specifically Berinert) dispensations were assumed to be for ODT due to dose size dispended and because SC pdC1INH is not reimbursed for LTP in Sweden. HAE, hereditary angioedema; IV, intravenous; LTP, long-term prophylaxis; n, number; ODT, on-demand treatment; pdC1INH, plasma-derived C1-esterase inhibitor; SC, subcutaneous.

Dispensation of lanadelumab and berotralstat started in 2020 and 2021, respectively, with lanadelumab dispensations showing a notable increase from 2021 to 2025 (**Table 2**). The annual number of new patients dispensing lanadelumab and berotralstat peaked in 2022, following their reimbursement in Sweden that year (31 and 15 patients, respectively) (**Table 2**). By May 2025 (data cut-off), one-third of the patients with HAE-specific medication dispensations in Sweden received LTP medication (71 patients [36%] dispensed lanadelumab; nine patients [5%] dispensed berotralstat) (**Table 2**).

**Table 2:** Prescription patterns of modern LTP therapies in Sweden: the number of unique new patients initiating modern LTP therapies lanadelumab or berotralstat per time period.

Modern LTP therapy	2020	2021	2022	2023	2024	January–May 2025
Lanadelumab	2	3	31	21	15	9
Berotralstat	0	1	15	3	6	2

The number of unique new patients (patients dispensing HAE-specific LTP therapy for the first time) initiating modern LTP therapies lanadelumab or berotralstat per time period. Patients can dispense more than one product per period. HAE, hereditary angioedema; IV, intravenous; LTP, long-term prophylaxis.

### 3.2 ODT dispensations among patients receiving modern LTP

To assess ODT dispensations among patients on LTP, ODT dispensation patterns were evaluated over each patient’s most recent 12 months of lanadelumab or berotralstat therapy corresponding to the recommended annual HAE treatment evaluation (1). The data showed that some patients dispensed one ODT product, whilst others dispensed multiple ODT products during the observation window.

#### 3.2.1 ODT dispensations during the most recent 12-months of LTP among patients receiving lanadelumab

We identified 81 patients with at least one lanadelumab dispensation between January 2020 and May 2025. Of these, 60 patients had lanadelumab dispensations for at least 12 months, while 21 had <12 months of lanadelumab dispensations and one patient had less than two dispensation events prior to the evaluation window and were excluded from the analysis (**Figure 1**). Of the 59 patients included in the analysis, the mean (standard deviation [SD]) duration of lanadelumab treatment (defined as the time interval between first and last dispensation) was 29.1 (10.01) months (median [range]; 29 months [12–57 months]).

Altogether, the 59 eligible patients had 200 ODT dispensation events during their most recent 12 months of lanadelumab treatment. For 49% (n=99/200) of these, information on the number of packages were available and indicated that one package was dispensed per event. Applying a scenario analysis, the number of packages was imputed for the remaining 51% (n=101/200) of the ODT dispensations where information on the number of packages was unavailable. The conservative scenario assumed patients received one package per imputed dispensation event, while the moderate scenario assumed two packages.

**Figure 2:**
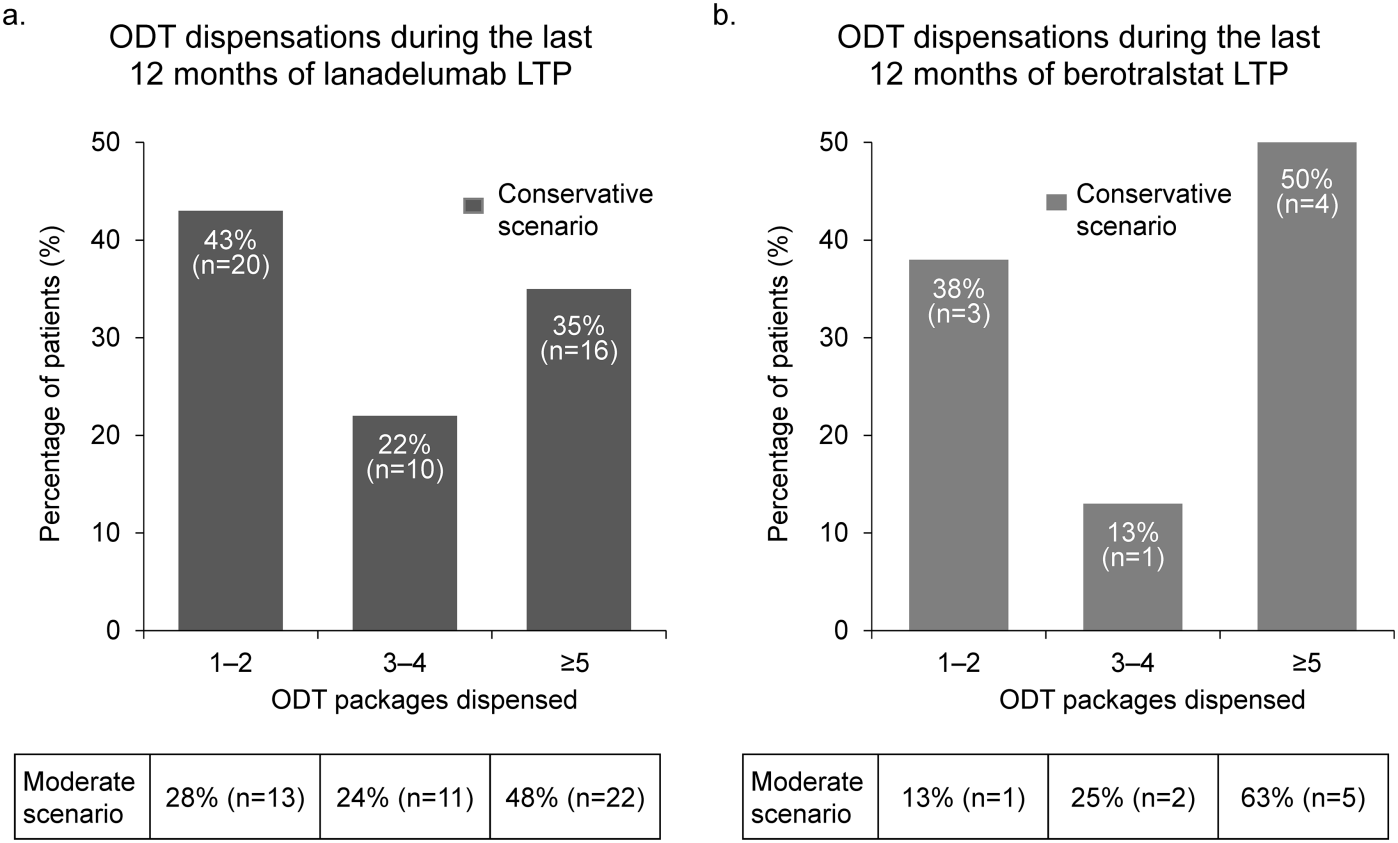
ODT dispensations among patients receiving lanadelumab or berotralstat LTP therapy. Proportions of patients dispensing 1–2, 3–4 and ≥5 packages of ODT medication within their individual last 12 months of **(A)** lanadelumab LTP or **(B)** berotralstat LTP. Plotted are the results from the conservative scenario which assumed one package of ODT medication was received per all dispensation events. The results from the moderate scenario, which assumed two packages per dispensation event are listed in the table below the chart. All ODT dispensations reported are in addition to any ODT stock dispensations. LTP, long-term prophylaxis; n, number; ODT, on-demand treatment.

Under the conservative scenario, 46 of 59 patients (78%) had at least one ODT dispensation event during their most recent 12 months of lanadelumab treatment. Of these, 20 out of 46 patients (43%) received 1–2 ODT packages, 10 (22%) received 3–4 packages, and 16 (35%) received ≥5 packages (range 5–29) during the annual period (**Figure 2A**). Among the 46 patients with ODT dispensations, the mean number of dispensed ODT packages was 4.3 per patient during their most recent 12-month period of lanadelumab. Importantly, these ODT dispensations occurred in addition to dispensation of the emergency stock medication patients should have at home (1).

Under the moderate scenario, 13 (28%) of the 46 patients with at least one ODT dispensation received 1–2 packages, 11 patients (24%) 3–4 packages and the proportion of patients with ≥5 ODT packages increased to 22 of 46 (48%), with package counts ranging from 5 to 58 (**Figure 2A**). In total, 13 out of 59 patients (22%) had no ODT dispensation events during their evaluation period.

Of the 81 patients dispensing lanadelumab during the overall study period, five patients stopped dispensing lanadelumab for unknown reasons, however they continued to dispense other HAE-specific medication until May 2025 (data cut). Of the five patients discontinuing lanadelumab, three patients continued to dispense ODT medication while two patients continued dispensing an alternative LTP medication (berotralstat) (data not shown). Out of those five patients, four patients dispensed lanadelumab for 1–3 months before switching to an alternative HAE-specific medication. The remaining one patient dispensed lanadelumab for 41 months before switching.

#### 3.2.2 ODT dispensations during the most recent 12-months of LTP among patients receiving berotralstat

A total of 27 patients had at least one berotralstat dispensation between January 2021 and May 2025. Of the 27 patients, nine had berotralstat dispensation for at least 12 months, while 18 had <12 months of treatment and were excluded from the analysis (**Figure 1**).

The mean (SD) duration of berotralstat treatment among the nine patients was 29.4 (11.28) months (median [range]; 31 months [12–48 months]). In total, there were 52 ODT dispensation events during the most recent 12-month period of berotralstat treatment. The number of packages was available for 22 out of 52 dispensation events (42%) revealing that one package was dispensed per dispensation event. Applying a scenario analysis, the number of packages was imputed for the remaining 58% (n=30/52) of the ODT dispensation events where information on the number of packages was unavailable. The same conservative and moderate scenarios assumed for lanadelumab were then applied to berotralstat.

Eight out of nine patients (89%) had at least one ODT dispensation event in addition to their emergency stock medication during their 12-month evaluation period. Three out of eight patients (38%) received 1–2 ODT packages, one patient (13%) received 3–4 packages, and four patients (50%) received ≥5 packages (range 5–15) during their most recent 12-months of berotralstat treatment. Among the eight patients with ODT dispensation, the mean number of dispensed ODT packages was 6.5 per patient during the 12-month evaluation period. In total, one out of nine patients (11%) had no ODT dispensation events during their evaluation period.

Under the moderate scenario, one (13%) of the eight patients with ODT dispensations received 1–2 packages, two patients (25%) 3–4 packages and the number of patients with ≥5 ODT packages increased to five of eight patients (63%), with package counts ranging from 6 to 19.

A total of 70% (19/27) of the patients initiating berotralstat treatment at any time during January 2021 until May 2025, stopped dispensing berotralstat LTP over the study period. Among these 19 patients, the mean (SD) duration of berotralstat treatment prior to discontinuation was 6.1 (5.78) months, with a median duration of three months (range 1–19). Overall, 16% (3/19) of patients who stopped berotralstat dispensations had received at least 12 months of treatment prior to discontinuation (mean [SD]; 16.3 months [3.79]).

Of the 19 patients who discontinued berotralstat, 11 (58%) switched to lanadelumab, the only alternative LTP treatment option available, while six (32%) discontinued berotralstat and continued to dispense ODT options. The remaining two patients (11%) initially received lanadelumab LTP before switching to berotralstat; both continued to dispense ODT, with one patient remaining on ODT options after discontinuing berotralstat and the other having no further dispensations of any HAE-specific medication after the last berotralstat dispensation event in the dataset until May 2025.

## 4 Discussion

This nationwide analysis of HAE-specific drug dispensations in Sweden highlights important trends in treatment patterns over the past five years. The steady increase in the annual number of unique patients receiving HAE-specific prescriptions suggests growing recognition and management of HAE within the healthcare system. The introduction and reimbursement of modern LTP options marked a significant change in therapeutic strategies underscoring the impact of access on treatment adoption. By May 2025, approximately one-third of patients in Sweden receiving HAE-specific medication were dispensing modern LTP therapy, with lanadelumab being the predominant choice. In addition to patients initiating modern LTP therapy over the observation time, several patients discontinued modern LTP and switched to alternative LTP or ODT treatment options. In the absence of clinical data to provide more insights, the reasons why patients discontinue modern LTP remain speculative and might relate to suboptimal treatment satisfaction potentially related to effectiveness, treatment burden, or tolerability in a subset of patients (11).

Although modern LTP therapy prescriptions are increasing, ODT options remain important as per guidelines’ recommendations and are widely dispensed alongside the availability of reimbursed modern LTP treatments, with the majority of patients still dispensing ODT throughout the study period. Contributing factors may include the high cost of LTP in a tax-funded healthcare system and restrictive reimbursement criteria, which limit eligibility to LTP and therefore access (8). Furthermore, ODT remains essential for managing breakthrough attacks in patients on modern LTP therapy not achieving total control of disease (1, 5, 6).

During the study time frame, dispensation of ODT medications was common in the group of patients with prescribed modern LTP therapy. The great majority of patients receiving lanadelumab or berotralstat LTP required at least one ODT dispensation in addition to their emergency stock medication, with a third or more patients requiring more than five ODT packages. These findings suggest that while some patients may achieve disease control with modern LTP agents, a substantial proportion of patients on LTP who are dispensing ODT medication may have residual disease activity, although this conclusion should be drawn with some caution, since ODT dispensation is an indirect proxy of disease control and LTP efficacy in this study. The observed requirement for ODT among a subset of patients, despite access to modern LTPs, may underscore variability in individual response and highlights the need for improved HAE care through novel and alternative prophylactic therapies (12). Additionally, the observed variability in LTP response and ODT dispensation reliance may be related to different HAE subtypes within the study population. Studies have shown that most patients have type 1 or 2 HAE for which suitable treatment options are available, and that HAE-n1C1INH is rare (4). However, we were unable to assess how subtype differences may have influenced ODT dispensation patterns as disease-specific data on HAE subtypes was not available in the registry data.

Several novel LTP therapies are currently in development, including prekallikrein inhibitors (ADX-324 and BW-20805), a KLKB1 gene editing therapy (Lonvo-Z), kallikrein inhibitors (STAR-0125 and ATN-249) and a bradykinin B2 receptor inhibitor (deucrictibant) (13). These agents have the potential to address unmet needs, reduce reliance on ODT, and offer alternative options for patients who respond inadequately to existing treatments. Notably, two subcutaneous agents— garadacimab, an activated factor XII inhibitor, and donidalorsen, a prekallikrein inhibitor—received European Commission approval in 2025 (14, 15). In phase 3 clinical trials, garadacimab administered once monthly reduced the mean number of attacks per month requiring ODT by 88%, whilst donidalorsen administered every four or eight weeks reduced the mean number of attacks per month requiring ODT by 92% and 67%, respectively, compared with placebo (14, 15). Emerging comparative evidence from a matching-adjusted indirect analysis suggests that patients receiving once-monthly garadacimab may experience a lower rate of ODT-requiring attacks than those treated with lanadelumab every two or four weeks; however, statistical significance was achieved only when comparing garadacimab with lanadelumab administered every four weeks (16). Ongoing evaluation will determine whether these novel therapies further transform the HAE treatment landscape and reduce reliance on ODT medication in clinical practice.

A key limitation of this study is that dispensed ODT and LTP medications were assumed rather than confirmed to have been administered. ODT dispensations do not necessarily indicate that an attack occurred or was treated, nor do they provide information on attack severity; therefore, ODT use was considered an indirect proxy for breakthrough attacks and potential limitations in LTP effectiveness. However, several factors not captured in this study—such as treatment adherence, dosing optimization, and barriers to access—may also influence LTP effectiveness. Consequently, ODT dispensations as an indirect proxy for LTP efficacy should be interpreted with caution. On the other hand, some attacks may go untreated altogether, which could result in an underestimation of attack frequency based on ODT dispensations. Although not considered standard practice, some ODT dispensations may be stockpiled by patients in case of breakthrough attacks, which could introduce a potential bias by overestimating reliance on ODT when dispensations are not directly linked to actual attacks. While we cannot completely rule out that some ODT dispensations were made to re-fill emergency stock, given that some patients did not make any ODT dispensations during the evaluation period, we consider this unlikely for the majority of the dispensations made. Additionally, information on ODT dispensation in the hospital setting was not available, potentially leading to an underestimation of overall ODT dispensation.

Although data for the number of packages dispensed were available for two out of the three ODT medications marketed in Sweden, some of the number of packages were based on imputed data which may result in the dispensations reported here to deviate from true values. Using a conservative imputation approach for missing package−size data ensures the study does not overestimate the utilization of ODT; if actual package sizes were larger than assumed, the true level of ODT may in fact be underestimated. Treatment discontinuation was based on dispensation data only, and information on the reasons for any treatment discontinuations were not captured in the study and should be interpreted with caution. All except for one patient discontinuing lanadelumab or berotralstat continued to dispense other HAE-specific medication, and none of these patients died or emigrated during the study period. A further limitation of the study is that HAE subtypes could not be distinguished, meaning that off-label use of HAE therapies in patients with normal C1 inhibitor cannot be excluded, although this subtype is rare and likely contributes minimally to the dataset. Any possible off-label dispensation of attenuated androgens and tranexamic acid, which has been reported in another study among a small proportion of patients (9) was not captured in the analysis and may have impacted the treatment patterns observed. Finally, there may also be the potential for misclassification of treatment utilization, as IV C1-INH dispensations may have been prescribed for prophylaxis rather than ODT.

In conclusion, this nationwide analysis provides important real-world insights into HAE treatment patterns in Sweden. The continued dispensation of ODT medication among patients receiving modern LTP therapy underscores ongoing unmet need, highlighting the importance of optimizing prophylactic strategies to achieve total disease control.

## Data Availability

The data analyzed in this study was obtained from the Swedish National Prescribed Drug Register, regulated by the Health data Register Act (1998:543) and the associated regulation (2005:363). Requests to access these datasets should be directed to the Swedish Board of Health and Welfare.
